# Supplementary material for: Unravelling reference bias in ancient DNA datasets
Source: Bioinformatics. 2024 Jul 3;40(7):btae436. doi: 10.1093/bioinformatics/btae436 (PMC11254355; doi:10.1093/bioinformatics/btae436)
Supplement: btae436_Supplementary_Data [file btae436_supplementary_data.zip › S1-S4_data_plots.zip!/S1-S4_data/mapping_stats_plots.zip!/mapping_stats_plots/local_bowtie_elephant_div0_10end_mapping_stats_byMQ_plots.pdf]

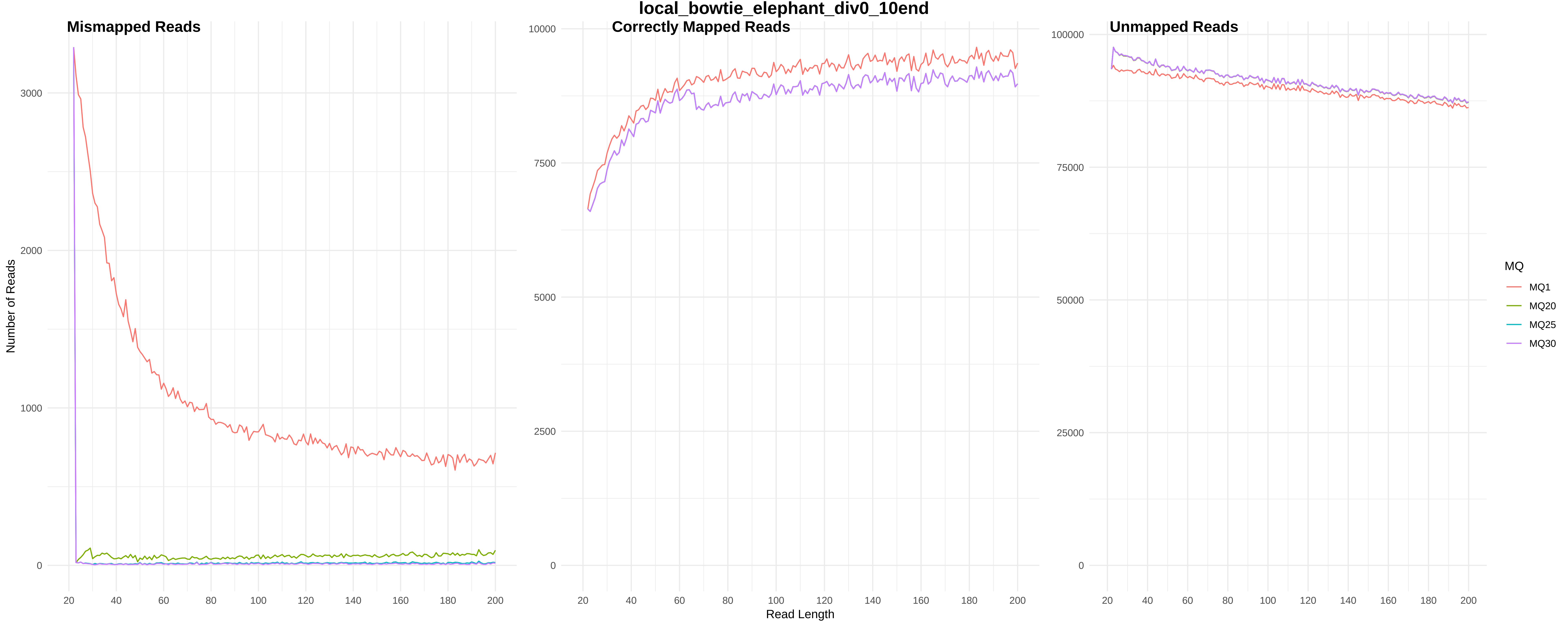

local_bowtie_elephant_div0_10end
Mismapped Reads
Correctly Mapped Reads
Unmapped Reads
Number of Reads
Read Length
3000
2000
1000
0
10000
7500
5000
2500
100000
75000
50000
25000
20
40
60
80
100
120
140
160
180
200
MQ
MQ1
MQ20
MQ25
MQ30
